# A new species, new host records and life cycle data for lepocreadiids (Digenea) of pomacentrid fishes from the Great Barrier Reef, Australia

**DOI:** 10.1007/s11230-022-10034-8

**Published:** 2022-04-08

**Authors:** Berilin Duong, Scott C. Cutmore, Thomas H. Cribb, Kylie A. Pitt, Nicholas Q.-X. Wee, Rodney A. Bray

**Affiliations:** 1 School of Biological Sciences, The University of Queensland, St Lucia, QLD 4072 Australia; 2 School of Environment and Science and Australian Rivers Institute, Griffith University, Gold Coast Campus, Gold Coast, QLD 4222 Australia; 3 Department of Life Sciences, Natural History Museum, Cromwell Road, London, SW7 5BD United Kingdom

## Abstract

A new species of lepocreadiid, *Opechonoides opisthoporus* **n. sp.**, is described infecting 12 pomacentrid fish species from the Great Barrier Reef, Australia, with *Abudefduf whitleyi* Allen & Robertson as the type-host. This taxon differs from the only other known member of the genus, *Opechonoides gure* Yamaguti, 1940, in the sucker width ratio, cirrus-sac length, position of the testes, position of the pore of Laurer’s canal, and relative post-testicular distance. The new species exhibits stenoxenic host-specificity, infecting pomacentrids from seven genera: *Abudefduf* Forsskål, *Amphiprion* Bloch & Schneider, *Neoglyphidodon* Allen, *Neopomacentrus* Allen, *Plectroglyphidodon* Fowler & Ball, *Pomacentrus* Lacépède and *Stegastes* Jenyns. Phylogenetic analyses of 28S rDNA sequence data demonstrate that *O. opisthoporus* **n. sp.** forms a strongly supported clade with *Prodistomum orientale* (Layman, 1930) Bray & Gibson, 1990. The life cycle of this new species is partly elucidated on the basis of ITS2 rDNA sequence data; intermediate hosts are shown to be three species of Ctenophora. New host records and molecular data are reported for *Lepocreadium oyabitcha* Machida, 1984 and *Lepotrema amblyglyphidodonis* Bray, Cutmore & Cribb, 2018, and new molecular data are provided for *Lepotrema acanthochromidis* Bray, Cutmore & Cribb, 2018 and *Lepotrema adlardi* (Bray, Cribb & Barker, 1993) Bray & Cribb, 1996. Novel *cox*1 mtDNA sequence data showed intraspecific geographical structuring between Heron Island and Lizard Island for *L. acanthochromidis* but not for *L. adlardi* or *O. opisthoporus* **n. sp.**

## Introduction

The Lepocreadiidae Odhner, 1905 is the largest family of the Lepocreadioidea Odhner, 1905, a group of digeneans primarily infecting fishes of shallow marine systems (Bray & Cribb, 2012). Eleven species in three lepocreadiid genera have been reported from the Pomacentridae (Table 1): *Lepocreadium* Stossich, 1904 (three species), *Lepotrema* Ozaki, 1932 (five species) and *Preptetos* Pritchard, 1960 (three species). Of these eleven species, it is likely that the infections of *Lepocreadium album* (Stossich, 1890) Stossich, 1904, *Preptetos cannoni* Barker, Bray & Cribb, 1993, *P. trulla* (Linton, 1907) Bray & Cribb, 1996, and *P. xesuri* (Yamaguti, 1940) Pritchard, 1960 are incidental infections, as each of these species has been reported primarily from fishes in other families: *L. album* in the Sparidae (Bartoli et al., 2005), *P. cannoni* in the Siganidae (Barker et al., 1993; Bray et al., 2022), *P. trulla* in the Lutjanidae (Claxton et al., 2017) and *P. xesuri* in the Acanthuridae (Bray et al., 1993; Bray & Cribb, 1996). On the Great Barrier Reef (GBR), there are reports of five lepocreadiid species occurring in pomacentrids (excluding infections of *Preptetos* spp.): *Lepotrema acanthochromidis* Bray, Cutmore & Cribb, 2018, *L. adlardi* (Bray, Cribb & Barker, 1993) Bray & Cribb, 1996, *L. amblyglyphidodonis* Bray, Cutmore & Cribb, 2018, *L. monile* Bray & Cribb, 1998, and *Lepocreadium oyabitcha* Machida, 1984. Additionally, there is a report of an undescribed *Lepotrema* species from the banded scalyfin, *Parma polylepis* Günther (as *Lepotrema* sp. 4 in Bray et al., 2018b).

**Table 1:** Species of lepocreadiids reported from pomacentrid fishes including information on host-specificity

Species	Host-specificity	Host and locality	References
*Lepocreadium album* (Stossich, 1890) Stossich, 1904	Euryxenic	*Chromis chromis* (Linnaeus) Saronic Gulf, Greece	Papoutsoglou (1976)
		Blenniidae Rafinesque; Centracanthidae Gill; Sparidae Rafinesque	See Bartoli et al. (2005)
*Lepocreadium oyabitcha* Machida, 1984	Stenoxenic	*Abudefduf vaigiensis* (Quoy & Gaimard) Off Ryukyu Islands, Japan *Abudefduf whitleyi* Allen & Robertson Off Lizard Island, GBR, Australia	Machida (1984); Bray & Cribb (1998)
*Lepocreadium sogandaresi* Nahhas & Powell, 1971	Oioxenic	*Stegastes leucostictus* (Müller & Troschel) Gulf of Mexico	Nahhas & Powell (1971)
*Lepotrema acanthochromidis* Bray, Cutmore & Cribb, 2018	Oioxenic	*Acanthochromis polyacanthus* Bleeker Off Lizard and Heron Islands, GBR, Australia	Barker et al. (1993); Barker et al. (1994); Bray et al. (2009); Bray et al. (2018b)
*Lepotrema adlardi* (Bray, Cribb & Barker, 1993) Bray & Cribb, 1996	Oioxenic	*Abudefduf bengalensis* (Bloch) Off Lizard and Heron Islands, GBR, Australia Ningaloo Reef, WA, Australia	Bray et al. (1993); Barker et al. (1994); Bray et al. (2018b)
*Lepotrema amblyglyphidodonis* Bray, Cutmore & Cribb, 2018	Stenoxenic	*Amblyglyphidodon curacao* (Bloch) Off Heron Island, GBR, Australia *Amphiprion akindynos* Allen Off Heron Island, GBR, Australia	Bray et al. (1993); Barker et al. (1994); Bray et al. (2018b)
*Lepotrema clavatum* Ozaki, 1932	Euryxenic	*Dascyllus albisella* Gill Off Hawaii, United States	Pritchard (1963)
		Balistidae Rafinesque; Chaetodontidae Rafinesque; Monacanthidae Nardo; Paralichthyidae Regan; Pomacanthidae Jordan & Evermann	See Bray et al. (2018b)
*Lepotrema monile* Bray & Cribb, 1998	Stenoxenic	*Pomacentrus amboinensis* Bleeker Off Lizard Island, GBR, Australia *Pomacentrus chrysurus* Cuvier Off Lizard Island, GBR, Australia *Stegastes apicalis* (De Vis) Off Heron Island, GBR, Australia *Pomacentrus wardi* Whitley Off Heron Island, GBR, Australia	Bray et al. (1993); Barker et al. (1994); Bray & Cribb (1998); Sun et al. (2012); Bray et al. (2018b)
*Lepotrema* sp. 4 of Bray et al. (2018b)	Unknown	*Parma polylepis* Günther Off Heron Island, GBR, Australia	Bray et al. (1993); Barker et al. (1994); Bray et al. (2018b)
*Preptetos cannoni* Barker, Bray & Cribb, 1993	Stenoxenic	*Pomacentrus bankanensis* Bleeker Off Heron Island, GBR, Australia	Bray et al. (1993); Barker et al. (1994)
		Siganidae Richardson	See Barker et al. (1993) and Bray et al. (2022)
*Preptetos trulla* (Linton, 1907) Bray & Cribb, 1996	Stenoxenic	*Chromis multilineata* (Guichenot) Off Puerto Rico	Dyer et al. (1985)
		Labridae Cuvier; Lutjanidae Gill; Sparidae Rafinesque	See Claxton et al. (2017)
*Preptetos xesuri* (Yamaguti, 1940) Pritchard, 1960	Stenoxenic	*Parma polylepis* Off Heron Island, GBR, Australia	Bray et al. (1993); Barker et al. (1994)
		Acanthuridae Bonaparte	See Bray & Cribb (1996) and Bray et al. (2022)

Pomacentrid hosts are listed in full with locality information and other taxa are listed at family level

*Abbreviations*: GBR, Great Barrier Reef; WA, Western Australia

In this study, we describe a new species of lepocreadiid from GBR pomacentrid fishes using morphological and molecular data, and use molecular data to identify its second intermediate hosts. New hosts are reported for *L. amblyglyphidodonis* and *Lepocreadium oyabitcha*, and novel molecular data are reported for *Lepotrema acanthochromidis*, *L. adlardi*, and *Lepocreadium oyabitcha*.

## Material and methods

### Specimen collection

Pomacentrid fishes were collected at three Australian localities: off Heron Island, southern GBR (23°26'S, 151°54'E) and off Lizard Island, northern GBR (14°40'S, 145°28'E) and in Moreton Bay, southeast Queensland (27°24'S, 153°26'E). Fishes were collected by line fishing, spearfishing, anaesthetic (using a clove oil solution), and barrier netting, and were euthanised immediately prior to dissecting. The gastrointestinal tract of each fish was removed and examined for trematodes under a stereo-microscope; trematodes were collected, and the gut was re-examined after a gut-wash, following the protocols of Cribb & Bray (2010). Live trematodes were fixed in near boiling saline and immediately preserved in 80% ethanol.

Ctenophores were sampled along the east coast of Australia between Brisbane, Queensland and Hobart, Tasmania on the Research Vessel *Investigator* between the 8th and 27th of May 2021. Ctenophores were collected at night by towing a bongo net (mouth diameter = 0.7 m; mesh size = 500 μm) obliquely from the surface to approximately 30 m and back to the surface. The duration of each tow ranged from 10–15 minutes and tow speed ranged from 1–1.5 m^-s^. Ctenophores were immediately removed from the cod end, measured, and inspected for trematode metacercariae. Metacercariae were collected and preserved in 96% ethanol.

### Morphological analysis

Specimens for morphological examination were rinsed with distilled water, overstained with Mayer’s haematoxylin, destained in a 1% hydrochloric acid solution and neutralised in a 1% ammonium hydroxide solution. Specimens were then dehydrated in a graded series of ethanol solutions (50%, 70%, 80%, 90%, 95% and twice in 100%) and cleared in methyl salicylate, before being mounted on glass slides in Canada balsam. Morphometric data were taken using a camera (Olympus SC50) mounted on a compound microscope (Olympus BX-53) and cellSens Standard imaging software. Measurements are in micrometres and are presented as a range, where length is followed by width, with the mean in parentheses. Drawings were made using a drawing tube attachment and digitised in Adobe Illustrator. Type- and voucher specimens are lodged in the Queensland Museum (QM), Brisbane, Australia, and the Natural History Museum (NHMUK), London, United Kingdom. To comply with the guidelines set out in article 8.5 of the amended 2012 version of the International Code of Zoological Nomenclature (ICZN, 2012), details of the new species have been submitted to ZooBank and registered with a Life Science Identifier (LSID), which is provided in the taxonomic summary.

### Molecular sequencing

Specimens for molecular analyses were either prepared as hologenophores, whereby a portion of the trematode is used for DNA sequencing and the remainder is used as a morphological voucher, or as paragenophores, whereby the trematode used for DNA sequencing is collected from the same individual host as the morphological voucher (Pleijel et al., 2008). Genomic DNA was extracted using a standard phenol/chloroform extraction method (Sambrook & Russell, 2001) and sequence data were generated for two ribosomal DNA (rDNA) markers, the large ribosomal subunit RNA coding region (28S) and the second internal transcribed spacer region (ITS2), and one mitochondrial DNA (mtDNA) marker, the cytochrome c oxidase subunit 1 (*cox*1). These regions were amplified using the following primers: LSU5 (5′-TAG GTC GAC CCG CTG AAY TTA AGC-3′, Littlewood, 1994) and 1500R (5′-GCT ATC CTG AGG GAA ACT TCG-3′, Snyder & Tkach, 2001) for 28S, 3S (5′-GGT ACC GGT GGA TCA CGT GGC TAG TG-3′, Morgan & Blair, 1995) and ITS2.2 (5′-CCT GGT TAG TTT CTT TTC CTC CGC-3′, Cribb et al., 1998) for ITS2, and Dig_cox1Fa (5′-ATG ATW TTY TTY TTY YTD ATG CC-3′, Wee et al., 2017) and Dig_cox1R (5′-TCN GGR TGH CCR AAR AAY CA AA-3′, Wee et al., 2017) for *cox*1.

A polymerase chain reaction (PCR) for each region was performed with a total of 20 μl comprising of 2 μl of DNA template for ITS2 or 4 μl for *cox*1 and 28S, 5 μl of MyTaq Reaction Buffer (Bioline), 0.75 μl of each primer for ITS2 and 28S or 2 μl for *cox*1, 0.25 μl of Taq DNA polymerase (Bioline MyTaq^TM^ DNA Polymerase) and made up with Invitrogen^TM^ ultraPURE^TM^ distilled water. A TaKaRa PCR Thermal Cycler was used to amplify each region using the following profiles: 28S: an initial 95°C denaturation for 4 minutes, 30 cycles of 95°C denaturation for 1 minute, 56°C annealing for 1 minute, 72°C extension for 2 minutes, 95°C denaturation for 1 minute, 55°C annealing for 45 seconds and a final 72°C extension for 4 minutes; ITS2: an initial 95°C denaturation for 3 minutes, 45°C annealing for 2 minutes, 72°C extension for 90 seconds, four cycles of 95°C denaturation for 45 seconds, 50°C annealing for 45 seconds, 72°C extension for 90 seconds, 30 cycles of 95°C denaturation for 20 seconds, 52°C annealing for 20 seconds, 72°C extension for 90 seconds and a final 72°C extension for 5 minutes; *cox*1: an initial 94°C denaturation for 3 minutes, 40 cycles of 94°C denaturation for 30 seconds, 50°C annealing for 30 seconds, 72°C extension for 30 seconds and a final 72°C extension for 10 minutes. Amplified DNA was sent to the Australian Genome Research Facility for purification and dual direction Sanger sequencing using the amplification primers for the ITS2 and *cox*1 regions, and the internal primers 300F (5′-CAA GTA CCG TGA GGG AAA GTT-3′, Littlewood et al., 2000) and ECD2 (5′-CTT GGT CCG TGT TTC AAG ACG GG-3′, Littlewood et al., 1997) for the 28S region. Sequences were assembled and edited in Geneious Prime version 2021.11.0.9 (https://www.geneious.com).

### Phylogenetic analyses

Newly generated ITS2 rDNA and *cox*1 mtDNA sequences were aligned with sequences of other lepocreadiid taxa available on GenBank (Table 2) in MEGA X (Kumar et al., 2018) using MUSCLE, with UPGMA clustering for iterations 1 and 2. The *cox*1 alignment was translated (echinoderm/flatworm mitochondrial code) and examined in Mesquite version 3.61 (Maddison & Maddison, 2021) for internal stop codons and to determine the correct reading frame. The alignment was trimmed after the correct reading frame was determined. All codon positions were then tested for non-stationarity in PAUP* version 4.0a (Swofford, 2003), and substitution saturation using the “Test of substitution saturation by Xia et al.” function (Xia et al., 2003; Xia & Lemey, 2009) implemented in DAMBE version 7.2 (Xia, 2018); non-stationarity and substitution saturation were not detected, and as such, all codons were used in subsequent analyses. Neighbour-joining analyses were conducted for each alignment with the following parameters: “Test of Phylogeny = Bootstrap method”, “No. of Bootstrap Replications = 10,000”, “Model/Method = No. of differences”, “Substitutions to Include = d: Transitions + Transversions”, “Rates among Sites = Uniform rates” and “Gaps/Missing Data Treatment = Pairwise deletion”. Pairwise differences for each alignment were estimated using the following parameters: “Variance Estimation Method = None”, “Model/Method = No. of differences”, “Substitutions to Include = d: Transitions + Transversions”, “Rates among Sites = Uniform rates” and “Gaps/Missing Data Treatment = Pairwise deletion”.

**Table 2:** Sequence data for species of *Lepocreadium*, *Lepotrema* and *Opechonoides* analysed in this study (GenBank accession numbers for *cox*1 mtDNA and ITS2 rDNA reference sequences and sequences generated in the present study, and host information)

Species	Host	Locality	GenBank accession #		References
			*cox*1 mtDNA	ITS2 rDNA	
*Lepocreadium oyabitcha* Machida, 1984	*Abudefduf sexfasciatus* (Lacépède)	LI	OM791389	OM777007	Present study
	*Abudefduf sordidus* (Forsskål)	LI	OM791390	OM777008	Present study
	*Abudefduf whitleyi* Allen & Robertson	LI	OM791391	OM777009	Present study
*Lepotrema acanthochromidis* Bray, Cutmore & Cribb, 2018	*Acanthochromis polyacanthus* (Bleeker)	HI LI	MH730025 OM791392 OM791393 OM791394 OM791395	MH729999 OM777010 OM777011	Bray et al. (2018b) Present study Present study
*Lepotrema adlardi* (Bray, Cribb & Barker, 1993) Bray & Cribb, 1996	*Abudefduf bengalensis* (Bloch)	HI LI	MH730027 MH730028 OM791396 OM791397 OM791398	MH730000	Bray et al. (2018b) Bray et al. (2018b) Present study
*Lepotrema amansis* Bray, Cutmore & Cribb, 2018	*Amanses scopas* (Cuvier)	HI	MH730029 MH730030 MH730031 MH730032		Bray et al. (2018b)
*Lepotrema amblyglyphidodonis* Bray, Cutmore & Cribb, 2018	*Amphiprion akindynos* Allen	HI	MH730033	MH730002	Bray et al. (2018b)
	*Amblyglyphidodon curacao* (Bloch)	HI	MH730034 MH730035	MH730003	Bray et al. (2018b)
	*Stegastes apicalis* (De Vis)	HI	OM791399		Present study
	*Plectroglyphidodon dickii* (Liénard)	HI	OM791400	OM777012	Present study
*Lepotrema cirripectis* Bray, Cutmore & Cribb, 2018	*Cirripectis chelomatus* Williams & Maugé	HI	MH730036		Bray et al. (2018b)
	*Cirripectis filamentosus* (Alleyne & MacLeay)	HI LI	MH730037 MH730038 MH730039 MH730040 MH730041		Bray et al. (2018b) Bray et al. (2018b)
*Lepotrema hemitaurichthydis* Bray, Cutmore & Cribb, 2018	*Hemitaurichthys polylepis* (Bleeker)	PA FP	MH730042 MH730043 MH730044 MH730045		Bray et al. (2018b) Bray et al. (2018b)
*Lepotrema melichthydis* Bray, Cutmore & Cribb, 2018	*Melichthys vidua* (Richardson)	PA	MH730046 MH730047		Bray et al. (2018b)
*Lepotrema monile* Bray & Cribb, 1998	*Pomacentrus wardi* Whitley	HI	MH730048 MH730049	MH730009	Bray et al. (2018b)
*Lepotrema moretonense* Bray, Cutmore & Cribb, 2018	*Prionurus maculatus* Ogilby	MB	MH730051		Bray et al. (2018b)
	*Prionurus microlepidotus* Lacépède	MB	MH730052 MH730053 MH730054		Bray et al. (2018b)
	*Selenotoca multifasciata* (Richardson)	MB	MH730055		Bray et al. (2018b)
*Opechonoides opisthoporus* n. sp.	*Abudefduf septemfasciatus* (Cuvier)	HI	OM791405	OM777013	Present study
	*Abudefduf sexfasciatus*	HI	OM791401	OM777014	Present study
	*Abudefduf whitleyi*	LI	OM791403	OM777015	Present study
	*Pomacentrus chrysurus* Cuvier	HI	OM791402	OM777016	Present study
	*Pomacentrus moluccensis* Bleeker	HI	OM791404	OM777017	Present study

*Abbreviations*: FP, French Polynesia; HI, off Heron Island; LI, off Lizard Island; MB, Moreton Bay; PA, off Palau

Newly generated partial 28S rDNA sequences were aligned with sequences from GenBank of related lepocreadiids using MUSCLE version 3.7 (Edgar, 2004) through the CIPRES Portal (Miller et al., 2010) with UPGMB clustering for iterations 1 and 2. Using Mesquite, the alignment was then refined by trimming and removing indels (with three or more base pairs) affecting at least 5% of sequences. To estimate the best-fitting nucleotide substitution model for the dataset, the refined alignment was analysed in jModelTest 2.1.10 (Darriba et al., 2012). The model ‘GTR + I + Γ’ was predicted to be the best estimator by the corrected Akaike Information Criterion and Bayesian Information Criterion. Phylogenetic analysis of the 28S dataset were conducted using maximum likelihood and Bayesian inference analyses on the CIPRES Portal. The maximum likelihood analysis was run using RAxML version 8.2.12 (Stamatakis, 2014) with 1,000 bootstrap pseudoreplicates, and the Bayesian inference analysis was run using MrBayes version 3.2.7a (Ronquist et al., 2012) with the following parameters: “ngen = 10,000,000”, “nruns = 2”, “nchains = 4”, “samplefreq = 1,000”, “nst = 6”, “rates = invgamma”, “ngammacat = 4”, “ratepr = variable”, “sumt burnin value = 3,000”, “sump burnin value = 3,000” and “burninfrac = 0.3”. Species of the Aephnidiogenidae Yamaguti, 1934 and the Gorgocephalidae Manter, 1966 were designated as outgroup taxa (Table 3).

**Table 3:** Sequence data for the Lepocreadioidea taxa analysed in this study (GenBank accession numbers for 28S rDNA reference sequences and sequences generated in the present study, and host information)

Species	Host	GenBank accession #	References
**Lepocreadiidae Odhner, 1905**			
*Bianium arabicum* Sey, 1996	*Lagocephalus lunaris* (Bloch & Schneider)	MH157076	Bray et al. (2018a)
*Bianium plicitum* (Linton, 1928) Stunkard, 1931	*Torquigener pleurogramma* (Regan)	MH157066	Bray et al. (2018a)
*Clavogalea trachinoti* (Fischthal & Thomas, 1968) Bray & Gibson, 1990	*Trachinotus coppingeri* Günther	FJ788471	Bray et al. (2009)
*Deraiotrema platacis* Machida, 1982	*Platax pinnatus* (Linnaeus)	MN073841	Bray et al. (2019)
*Diplocreadium tsontso* Bray, Cribb & Barker, 1996	*Balistoides conspicillum* (Bloch & Schneider)	FJ788472	Bray et al. (2009)
*Diploproctodaeum momoaafata* Bray, Cribb & Barker, 1996	*Ostracion cubicum* Linnaeus	FJ788474	Bray et al. (2009)
*Diploproctodaeum monstrosum* Bray, Cribb & Justine, 2010	*Arothron stellatus* (Anonymous)	FJ788473	Bray et al. (2009)
*Echeneidocoelium indicum* Simha & Pershad, 1964	*Echeneis naucrates* Linnaeus	FJ788475	Bray et al. (2009)
*Hypocreadium lamelliforme* (Linton, 1907) Bravo Hollis & Manter, 1957	*Balistes capriscus* Gmelin, 1789	MZ345680	Curran et al. (2021)
*Hypocreadium* cf. *patellare* Yamaguti, 1938	*Balistoides viridescens* (Bloch & Schneider)	FJ788478	Bray et al. (2009)
*Hypocreadium picasso* Bray, Cribb & Justine, 2009	*Rhinecanthus aculeatus* (Linnaeus)	FJ788479	Bray et al. (2009)
*Hypocreadium toombo* Bray & Justine, 2006	*Pseudobalistes fuscus* (Bloch & Schneider)	FJ788480	Bray et al. (2009)
*Lepidapedoides angustus* Bray, Cribb & Barker, 1996	*Epinephelus cyanopodus* (Richardson)	FJ788482	Bray et al. (2009)
*Lepocreadium opsanusi* Sogandares & Hutton, 1960	*Umbrina xanti* Gill	MK648298	Pérez-Ponce de León & Hernández-Mena (2019)
*Lepocreadium oyabitcha* Machida, 1984	*Abudefduf sordidus* (Forsskål)	OM777006	Present study
*Lepotrema acanthochromidis* Bray, Cutmore & Cribb, 2018	*Acanthochromis polyacanthus* (Bleeker)	MH730014	Bray et al. (2018b)
*Lepotrema adlardi* (Bray, Cribb & Barker, 1993) Bray & Cribb, 1996	*Abudefduf bengalensis* (Bloch)	MH730015	Bray et al. (2018b)
*Lepotrema amblyglyphidodonis* Bray, Cutmore & Cribb, 2018	*Amphiprion akindynos* Allen	MH730017	Bray et al. (2018b)
*Lepotrema monile* Bray & Cribb, 1998	*Pomacentrus wardi* Whitley	MH730024	Bray et al. (2018b)
*Lobatocreadium exiguum* (Manter, 1963) Madhavi, 1972	*Pseudobalistes fuscus*	FJ788484	Bray et al. (2009)
*Mobahincia teirae* Bray, Cribb & Cutmore, 2018	*Platax teira* (Forsskål)	MH157068	Bray et al. (2018a)
*Multitestis magnacetabulum* Mamaev, 1970	*Platax teira*	FJ788485	Bray et al. (2009)
*Neohypocreadium dorsoporum* Machida & Uchida, 1987	*Chaetodon flavirostris* Günther	FJ788487	Bray et al. (2009)
*Neomultitestis aspidogastriformis* Bray & Cribb, 2003	*Platax teira*	FJ788489	Bray et al. (2009)
*Neopreptetos arusettae* Machida, 1982	*Pomacanthus sexstriatus* (Cuvier)	FJ788490	Bray et al. (2009)
*Opechona austrobacillaris* Bray & Cribb, 1998	*Pomatomus saltatrix* (Linnaeus)	MH157073	Bray et al. (2018a)
*Opechona chloroscombri* Nahhas & Cable, 1964	*Chloroscombrus chrysurus* (Linnaeus)	MZ345679	Curran et al. (2021)
*Opechona kahawai* Bray & Cribb, 2003	*Arripis trutta* (Forster)	FJ788491	Bray et al. (2009)
*Opechona corkumi* Curran, Martorelli & Overstreet, 2021	*Peprilus burti* Fowler	MZ345683	Curran et al. (2021)
*Opechona olssoni* (Yamaguti, 1934) Yamaguti, 1938	*Scomber japonicus* Houttuyn	MT303947	Sokolov et al. (2020)
*Opechonoides opisthoporus* **n. sp.**	*Abudefduf whitleyi* Allen & Robertson	OM777005	Present study
*Pelopscreadium spongiosum* (Bray & Cribb, 1998) Dronen, Blend, Khalifa, Mohamadain & Karer, 2016	*Ostracion cubicum*	FJ788469	Bray et al. (2009)
*Preptetos laguncula* Bray & Cribb, 1996	*Naso unicornis* (Forsskål)	MZ701988	Bray et al. (2021)
*Preptetos paracaballeroi* Bray, Cutmore & Cribb, 2022	*Naso annulatus* (Quoy & Gaimard)	MZ702003	Bray et al. (2021)
*Preptetos prudhoei* Bray, Cutmore & Cribb, 2022	*Zebrasoma scopas* (Cuvier)	MZ701995	Bray et al. (2021)
*Preptetos trulla* (Linton, 1907) Bray & Cribb, 1996	*Ocyurus chrysurus* (Bloch)	AY222237	Olson et al. (2003)
*Prodistomum alaskense* (Ward & Fillingham, 1934) Bray & Merrett, 1998	*Aptocyclus ventricosus* (Pallas)	MT303950	Sokolov et al. (2020)
*Prodistomum keyam* Bray & Cribb, 1996	*Monodactylus argenteus* (Linnaeus)	FJ788493	Bray et al. (2009)
*Prodistomum orientale* (Layman, 1930) Bray & Gibson, 1990	*Scomber japonicus*	MT299625	Sokolov et al. (2020)
*Prodistomum priedei* Bray & Merrett, 1998	*Epigonus telescopus* (Risso)	AJ405272	Bray et al. (1999)
**Aephnidiogenidae Yamaguti,1934**			
*Aephnidiogenes major* Yamaguti, 1934	*Diagramma labiosum* MacLeay	FJ788468	Bray et al. (2009)
*Austroholorchis sprenti* (Gibson, 1987) Bray & Cribb, 1997	*Sillago ciliata* Cuvier	MH157075	Bray et al. (2018a)
*Holorchis castex* Bray & Justine, 2007	*Diagramma pictum* (Thunberg)	FJ788476	Bray et al. (2009)
**Gorgocephalidae Manter, 1966**			
*Gorgocephalus graboides* Huston, Cutmore, Miller, Sasal, Smit & Cribb, 2021	*Kyphosus cinerascens* (Forsskål)	MW353905	Huston et al. (2021)
*Gorgocephalus yaaji* Bray & Cribb, 2005	*Kyphosus cinerascens*	KU951489	Huston et al. (2016)

## Results

### Overview

We have examined over 1,800 pomacentrids from Moreton Bay, off Heron Island and off Lizard Island since 1990 (Table 4). These specimens comprise 61 species from 16 genera: *Abudefduf* Forsskål, six species (570 individuals); *Acanthochromis* Gill, one species (169 individuals); *Amblyglyphidodon* Bleeker, two species (98 individuals); *Amphiprion* Bloch & Schneider, three species (28 individuals); *Chromis* Cuvier, six species (113 individuals); *Chrysiptera* Swainson, seven species (24 individuals); *Dascyllus* Cuvier, three species (117 individuals); *Dischistodus* Gill, three species (73 individuals); *Hemiglyphidodon* Bleeker, one species (five individuals); *Neoglyphidodon* Allen, two species (42 individuals); *Neopomacentrus* Allen, two species (32 individuals); *Parma* Günther, two species (9 individuals); *Plectroglyphidodon* Fowler & Bean, three species (31 individuals); *Pomacentrus* Lacépède, 15 species (429 individuals); *Premnas* Cuvier, one species (one individual); and *Stegastes* Jenyns, four species (128 individuals).

**Table 4:** Prevalence of *Opechonoides opisthoporus* **n. sp.** in Great Barrier Reef and Moreton Bay pomacentrid fishes

Genus	Species	Moreton Bay	Heron Island	Lizard Island
*Abudefduf*	*bengalensis* (Bloch)	0/65	**2/50**	0/10
	*septemfasciatus* (Cuvier)		**1/2**	**1/5**
	*sexfasciatus* (Lacépède)	0/3	**3/33**	**1/26**
	*sordidus* (Forsskål)	0/3		0/3
	*vaigiensis* (Quoy & Gaimard)	0/29	0/1	0/4
	*whitleyi* Allen & Robertson	0/29	**1/290**	**2/17**
*Acanthochromis*	*polyacanthus* (Bleeker)		0/80	0/89
*Amblyglyphidodon*	*curacao* (Bloch)		0/64	0/32
	*leucogaster* (Bleeker)		0/2	
*Amphiprion*	*akindynos* Allen		0/14	**1/2**
	*melanopus* Bleeker			0/2
	*perideraion* Bleeker		0/10	
*Chromis*	*amboinensis* (Bleeker)		0/1	
	*atripectoralis* Welander & Schultz		0/17	0/31
	*nitida* (Whitley)		0/10	
	*ternatensis* (Bleeker)			0/2
	*viridis* (Cuvier)		0/11	0/31
	*weberi* Fowler & Bean			0/10
*Chrysiptera*	*biocellata* (Quoy & Gaimard)		0/5	
	*cyanea* (Quoy & Gaimard)			0/6
	*flavipinnis* (Allen & Robertson)		0/3	
	*rex* (Snyder)			0/1
	*rollandi* (Whitley)			0/7
	*taupou* (Jordan & Seale)			0/1
	*unimaculata* (Cuvier)		0/1	
*Dascyllus*	*aruanus* (Linnaeus)		0/54	0/19
	*reticulatus* (Richardson)	0/1	0/17	0/24
	*trimaculatus* (Rüppell)	0/2		
*Dischistodus*	*melanotus* (Bleeker)		0/22	0/6
	*perspicillatus* (Cuvier)		0/24	0/11
	*pseudochrysopoecilus* (Allen & Robertson)		0/5	0/5
*Hemiglyphidodon*	*plagiometapon* (Bleeker)			0/5
*Neoglyphidodon*	*melas* (Cuvier)		0/14	**1/27**
	*nigroris* (Cuvier)			0/1
*Neopomacentrus*	*azysron* (Bleeker)			**3/30**
	*cyanomos* (Bleeker)			0/2
*Parma*	*oligolepis* Whitley	0/3		
	*polylepis* Günther		0/6	
*Plectroglyphidodon*	*dickii* (Liénard)		0/6	
	*lacrymatus* (Quoy & Gaimard)		0/6	0/12
	*leucozonus* (Bleeker)		0/5	**1/2**
*Pomacentrus*	*adelus* Allen			**2/33**
	*amboinensis* Bleeker	0/1	0/4	0/43
	*bankanensis* Bleeker		0/12	
	*brachialis* Cuvier	0/1	0/2	
	*chrysurus* Cuvier		**3/34**	**1/32**
	*coelestis* Jordan & Starks		0/1	0/16
	*lepidogenys* Fowler & Bean		0/8	
	*moluccensis* Bleeker		**2/58**	0/27
	*nagasakiensis* Tanaka		0/3	0/3
	*nigromarginatus* Allen		0/1	
	*pavo* (Bloch)			0/10
	*philippinus* Evermann & Seale		0/4	
	*tripunctatus* Cuvier		0/2	
	*vaiuli* Jordan & Seale		0/3	
	*wardi* Whitley	0/1	0/122	0/8
*Premnas*	*biaculeatus* (Bloch)			0/1
*Stegastes*	*apicalis* (De Vis)		**1/83**	0/29
	*fasciolatus* (Ogilby)		0/5	0/1
	*gascoynei* (Whitley)	0/1		
	*nigricans* (Lacépède)			0/9

Numbers for infected fish are shown in bold

We collected adult trematodes consistent with the Lepocreadiidae from pomacentrids off Heron Island and Lizard Island; no adult lepocreadiids were found in Moreton Bay pomacentrids. Of the pomacentrids collected since 1990, we found specimens of an unknown species of *Opechonoides* Yamaguti, 1940 in species of multiple pomacentrid genera off Heron Island and Lizard Island. In more recent collections (between July 2019 to April 2021, 662 individuals), new specimens of four known species of lepocreadiids were also collected. *Lepotrema acanthochromidis* and *L. adlardi* were collected from known hosts, *Acanthochromis polyacanthus* (Bleeker) and *Abudefduf bengalensis* (Bloch), respectively (off both Heron Island and Lizard Island). *Lepotrema amblyglyphidodonis* was collected from known hosts, *Amblyglyphidodon curacao* (Bloch) and *Amphiprion akindynos* Allen, and two new hosts, *Plectroglyphidodon dickii* (Liénard) and *Stegastes apicalis* (De Vis) off Heron Island. *Lepocreadium oyabitcha* was collected from a known host, *Abudefduf whitleyi* Allen & Robertson, and three new hosts, *A. bengalensis* (Bloch), *A. sexfasciatus* (Cuvier) and *A. sordidus* (Forsskål) at Lizard Island. Prevalence data in taxonomic summaries are based on new collections (July 2019 to April 2021) for all taxa except for the new species of *Opechonoides.*

Four ctenophore species were collected: *Hormiphora* (?) sp. (20 individuals), *Ocyropsis* (?) sp. (32 individuals) and *Pukia falcata* Gershwin, Zeidler & Davie (32 individuals) over the continental shelf of southeast Queensland and *Bolinopsis* (?) sp. (20 individuals) off the east coast of Tasmania. Metacercariae consistent with the Lepocreadiidae were collected from *Bolinopsis* sp., *Ocyropsis* sp. and *P. falcata*. Although we are confident that three species of ctenophore were infected, only *P. falcata* could be identified reliably to species. The other two species were unidentifiable due to the combination of damage to the specimens at the time of collection, inability to generate informative DNA sequences, and the unsettled nature of the taxonomy of ctenophores in Australian waters. As such, on the basis of their general morphology, we tentatively identified these specimens as relating to *Bolinopsis* and *Ocyropsis*. Unfortunately, the difficulties of preservation of ctenophores precludes the lodgement of morphologically informative voucher specimens in a museum.

### Molecular results

**Fig. 1:**
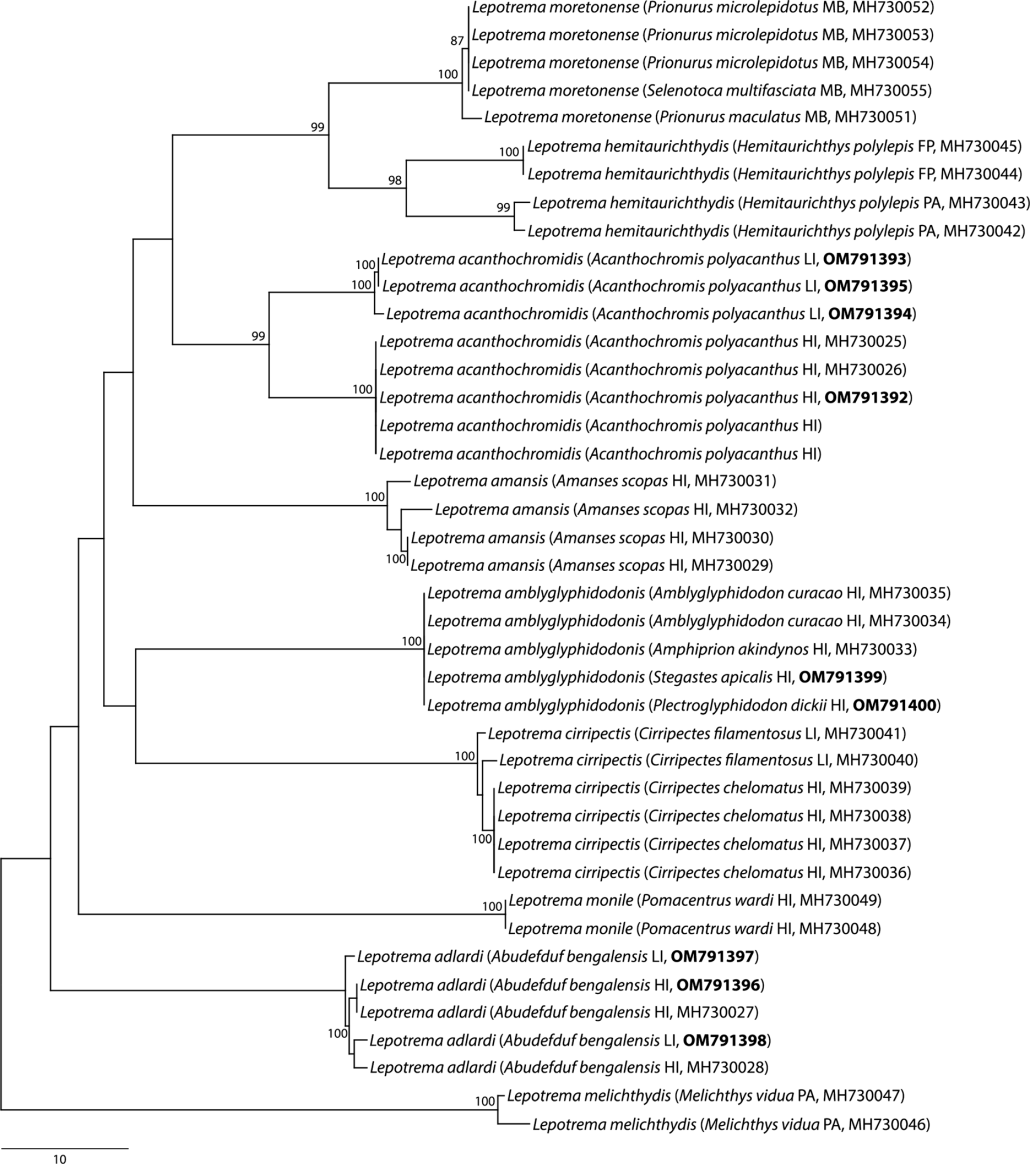
Phylogram from the unrooted neighbour-joining analysis of the *cox*1 mtDNA dataset for the genus *Lepotrema* (Lepocreadiidae). The newly generated sequences are shown in bold. Bootstrap support values are shown at the nodes; support values below 80 are not shown. *Abbreviations*: FP, French Polynesia; HI, off Heron Island; LI, off Lizard Island; MB, Moreton Bay; PA, Palau. The scale bar indicates the number of base differences

ITS2 sequence data were generated for the new species of *Opechonoides*, *Lepotrema acanthochromidis*, *L. amblyglyphidodonis*, *Lepocreadium oyabitcha* and the lepocreadiid metacercariae. No intraspecific genetic variation was present for any of the species examined. The ITS2 sequences of the metacercariae collected from the ctenophores were identical to that of the new species of *Opechonoides*. *cox*1 data were generated for the new species of *Opechonoides*, *Lepotrema acanthochromidis*, *L. adlardi*, *L. amblyglyphidodonis* and *Lepocreadium oyabitcha*. Newly generated sequences of *Lepotrema acanthochromidis* from off Heron Island were identical to those available on GenBank, also from off Heron Island, and differed from sequences of Lizard Island specimens by up to 18 base pairs, showing clear geographical structuring (Fig. 1). In contrast, the *cox*1 sequences of *L. adlardi* from off Heron Island and Lizard Island differed by up to only two base pairs and showed no geographical structuring. New *cox*1 sequences of *L. amblyglyphidodonis* from the two new hosts from off Heron Island were identical to those collected from *Amblyglyphidodon curacao* and *Amphiprion akindynos* (available on GenBank and also from off Heron Island). The *cox*1 sequences for specimens of *Lepocreadium oyabitcha* and the new species of *Opechonoides* showed no intraspecific genetic variation.

**Family Lepocreadiidae Odhner, 1905**

Genus ***Opechonoides*** Yamaguti, 1940

*Type-species*: *Opechonoides gure* Yamaguti, 1940, by original designation.

***Opechonoides opisthoporus*** **n. sp.**

*Type-host*: *Abudefduf whitleyi* Allen & Robertson, Whitley’s sergeant (Pomacentridae).

*Type-locality*: Off Lizard Island, northern Great Barrier Reef, Australia.

*Other hosts*: *Abudefduf bengalensis* (Bloch), Bengal sergeant; *A. septemfasciatus* (Cuvier), Banded sergeant; *A. sexfasciatus* (Lacépède), Scissortail sergeant; *Amphiprion akindynos* Allen, Barrier Reef anemonefish; *Neoglyphidodon melas* (Cuvier), Bowtie damsel; *Neopomacentrus azysron* (Bleeker), Yellowtail demoiselle; *Plectroglyphidodon leucozonus* (Bleeker), Whiteband damsel; *Pomacentrus adelus* Allen, Obscure damsel; *P. chrysurus* Cuvier, Whitetail damsel; *P. moluccensis* Bleeker, Lemon damsel; *Stegastes apicalis* (De Vis), Australian gregory (Pomacentridae).

*Other locality*: Off Heron Island, southern Great Barrier Reef, Australia.

*Site in host*: Intestine.

*Abundance and prevalence*: Off Lizard Island: four specimens from one of five *A. septemfasciatus*; two specimens from one of 26 *A. sexfasciatus*; eight specimens from two of 17 *A. whitleyi*; two specimens from one of two *A. akindynos*; one specimen from one of 27 *N. melas*; seven specimens from three of 30 *N. azysron*; one specimen from one of two *P. leucozonus*; four specimens from two of 33 *P. adelus*; one specimen from one of 32 *P. chrysurus*. Off Heron Island: two specimens from two of 50 *A. bengalensis*; nine specimens from one of two *A. septemfasciatus*; six specimens from three of 33 *A. sexfasciatus*; six specimens from one of 290 *A. whitleyi*; four specimens from three of 34 *P. chrysurus*; two specimens from two of 58 *P. moluccensis*; one specimen from one of 83 *S. apicalis*.

*Type-material*: Holotype (QM G240016) and 28 paratypes (QM G240017–42, NHMUK 2022.2.25.1–2), including five hologenophores.

*Representative DNA sequences*: ITS2 rDNA, five identical sequences; 28S rDNA, one sequence; *cox*1 mtDNA, five identical sequences (See Tables 2 and 3 for GenBank accession numbers).

*ZooBank LSID*: urn:lsid:zoobank.org:act:1A4C1EB6-DE1D-4F2B-958C-0BD2AF3B7D9E.

*Etymology*: The epithet *opisthoporus* refers to the unusually posterior position of the dorsal pore of Laurer’s canal.

### Description (Fig. 2)

**Fig. 2:**
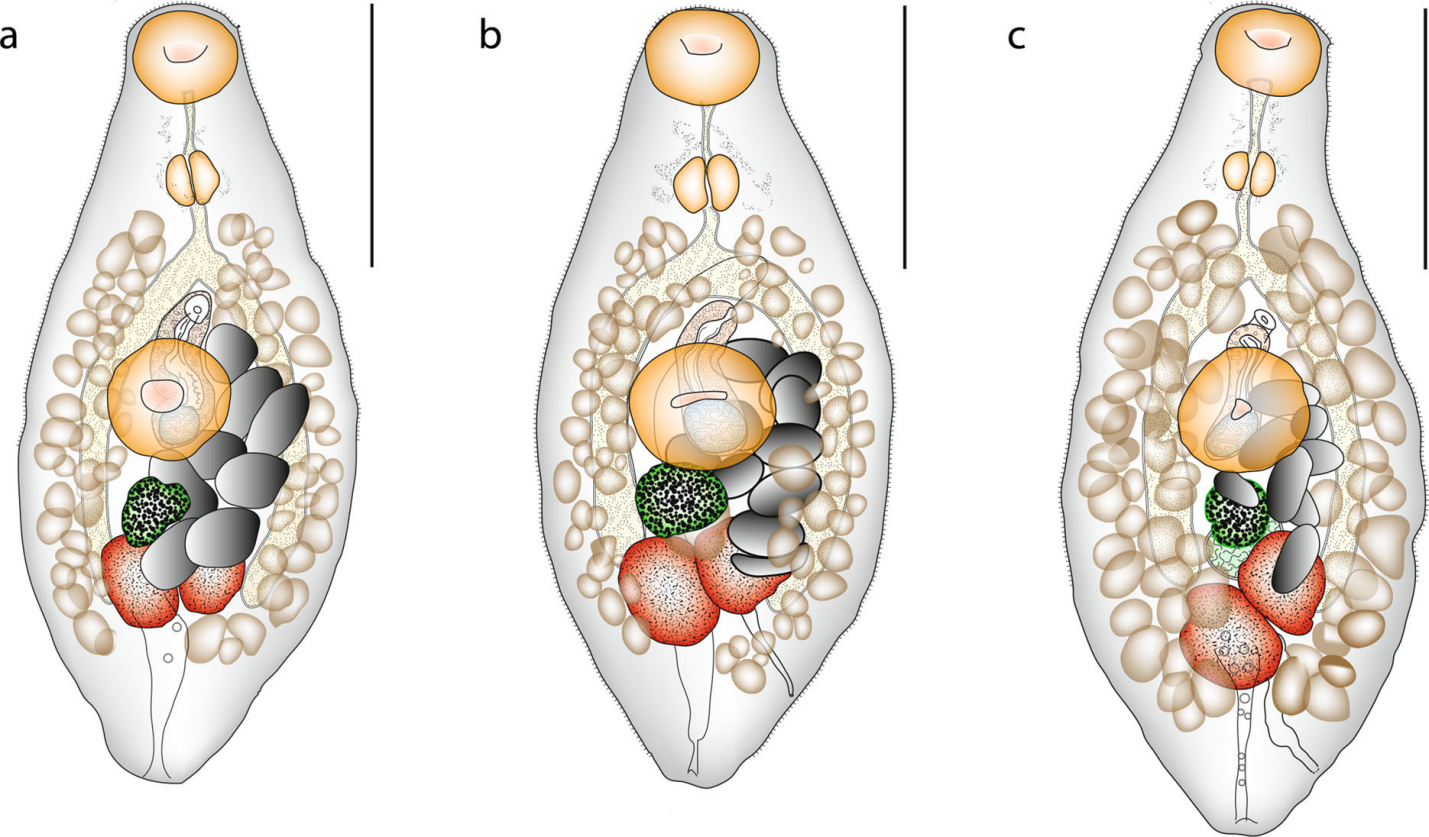
*Opechonoides opisthoporus* n. sp. from off Lizard Island, Great Barrier Reef, Australia; a holotype ex *Abudefduf whitleyi* ventral view; b paratype ex *Abudefduf septemfasciatus* ventral view; c paratype ex *Amphiprion akindynos* ventral view. Scale bars: 200 µm

[Based on 24 whole mounts and five hologenophores.] Body small, fusiform, 502–706 × 233–324 (581 × 256). Tegument spined; extent of spines variable, to anterior hindbody, level of testes or close to posterior extremity. Eye-spot pigment scattered widely around prepharynx and pharynx. Oral sucker transversely oval, subterminal, 65–89 × 75–97 (72 × 85). Ventral sucker rounded, 84–121 × 94–115 (100 × 104), in mid-region of body. Prepharynx distinct, 20–40 (33), narrow. Pharynx oval, 37–50 × 42–53 (40 × 44). Oesophagus distinct, 14–34 (24). Intestinal bifurcation in posterior forebody. Caeca short, terminating at level of testes. Testes two, oval, entire, oblique, in mid-hindbody, 47–92 × 47–92 (66 × 65). External seminal vesicle not detected. Cirrus-sac claviform or dumbbell-shaped, 64–164 × 31–50 (118 × 36). Internal seminal vesicle oval. Pars prostatica vesicular, lined with anuclear cell-like bodies. Ejaculatory duct long. Genital atrium distinct. Genital pore sinistrally submedian, post-bifurcal, in posterior forebody. Ovary entire or slightly indented, 34–59 × 34–65 (40 × 44), often obscured by eggs. Laurer’s canal runs diagonally from dorsal to testes to sinistrally submarginal dorsal pore close to posterior extremity. Uterus from mid-testicular level to ventral sucker, mostly intracaecal. Eggs few, large, tanned, operculate, 30–49 × 57–79 (35 × 67). Metraterm muscular, short. Vitellarium follicular; follicles relatively large, with fields reaching from anterior oesophagus to anterior post-testicular region, not reaching close to posterior extremity. Excretory pore dorsally sub-terminal; vesicle narrow posteriorly, widens and reaches to intestinal bifurcation or just anterior, often contains corpuscles in highly characteristic single column.

### Remarks

The features that suggest that this new species belongs in *Opechonoides* include the possession of a uterus that overlaps the testes, short caeca, a long excretory vesicle containing corpuscles, and oblique testes. It differs from the only other species of the genus, *O. gure* Yamaguti, 1940, in having a greater ventral to oral sucker width ratio (1:1.18–1.25 *vs* 1:0.80), a shorter cirrus-sac (118 μm *vs* 190 μm), contiguous testes (*vs* separated), and a longer post-testicular distance (14–19% *vs* 6% of body length) with a distinct posterior region lacking vitelline follicles. Additionally, the position and course of Laurer’s canal is unusual and not exactly as described for *O. gure*. In the latter species, Laurer’s canal does not reach the posterior extremity, but extends only to the level of the posterior testis, and opens ‘a little to the left of the median line’, not submarginally. Molecular data were generated for specimens collected from five of the 12 host species; the remaining specimens were identified based on morphology.

### Intermediate hosts

*Second intermediate hosts*: *Bolinopsis* (?) sp. (Bolinopsidae); *Ocyropsis* (?) sp. (Ocyropsidae); *Pukia falcata* Gershwin, Zeidler & Davie (Pukiidae).

*Localities*: Off the continental shelf of southeast Queensland, Australia; off the east coast of Tasmania, Australia (see Table 5).

**Table 5:** Second intermediate host information (ctenophore species, size, locality and ITS2 rDNA GenBank accession numbers) for *Opechonoides opisthoporus* **n. sp.**

Host species	Diameter (mm)	Locality	Latitude	Longitude	GenBank accession #
*Bolinopsis* sp.	–	TAS	42°36'S	148°16'E	OM777004
*Ocyropsis* sp.	–	SE QLD	27°17'S	153°35'E	OM776998
*Ocyropsis* sp.	–	SE QLD	26°50'S	153°32'E	OM776999
*Ocyropsis* sp.	–	SE QLD	26°50'S	153°31'E	OM777000
*Ocyropsis* sp.	–	SE QLD	26°42'S	153°41'E	OM777001
*Pukia falcata* Gershwin, Zeidler & Davie	13	SE QLD	26°23'S	153°47'E	OM777002
*P. falcata*	14	SE QLD	25°31'S	153°48'E	OM777003

*Abbreviations*: SE QLD, off the coast of southeast Queensland; TAS, off the east coast of Tasmania

*Site of infection*: Mesogloea.

*Representative DNA sequences*: ITS2 rDNA, seven identical sequences (see Table 5 for GenBank accession numbers).

### Remarks

Metacercariae recovered from three ctenophore species were genetically matched to *O. opisthoporus ***n. sp.** on the basis of identical ITS2 sequences. No morphological vouchers were available for examination as all specimens were used for molecular sequencing due to their small size.

Genus ***Lepocreadium*** Stossich, 1904

*Type-species*: *Lepocreadium album* (Stossich, 1890) Stossich, 1904, by original designation.

***Lepocreadium oyabitcha*** Machida, 1984

*Type*-*host*: *Abudefduf vaigiensis* (Quoy & Gaimard), Indo-Pacific sergeant (Pomacentridae).

*Type*-*locality*: Off Okinawa, Japan.

*Other host*: *Abudefduf whitleyi* Allen & Robertson, Whitley’s sergeant (Pomacentridae).

*Other locality*: Off Lizard Island, northern Great Barrier Reef, Australia.

*Records*: Machida (1984); Bray & Cribb (1998).

### New material

*New hosts: Abudefduf bengalensis* (Bloch), Bengal sergeant; *A. sexfasciatus* (Cuvier), Scissortail sergeant; *A. sordidus* (Forsskål), Blackspot sergeant (Pomacentridae).

*Known host*: *A. whitleyi*.

*Known locality*: Off Lizard Island.

*Site in host*: Gall bladder.

*Abundance and prevalence*: Two specimens from two of 10 *A. bengalensis*; 21 specimens from eight of 26 *A. sexfasciatus*; five specimens from one of three *A. sordidus*; 18 specimens from six of 15 *A. whitleyi*.

*Voucher material*: Six specimens (QM G240043–8), including three hologenophores.

*Representative DNA sequences*: ITS2 rDNA, five identical sequences (three submitted to GenBank); 28S rDNA, one sequence; *cox*1 mtDNA, five identical sequences (three submitted to GenBank; see Tables 2 and 3 for GenBank accession numbers).

### Remarks

Morphologically, the new specimens closely resemble the descriptions of Machida (1984) and Bray & Cribb (1998), especially in the distinctive tri-lobed ovary and the ramifying vitellarium. All specimens were collected from the gall bladder, which is consistent with the original report of Machida (1984); Bray & Cribb (1998) reported their specimen from the intestine, but we suspect that the worm had been relocated to the intestine from the gall bladder during dissection. Although Machida (1984) reported this worm to occur in pairs, Bray & Cribb (1998) reported a single worm from an individual host, and in the present study, up to five specimens were recovered from a single host.

Genus ***Lepotrema*** Ozaki, 1932

*Type-species*: *Lepotrema clavatum* Ozaki, 1932, by original designation.

***Lepotrema acanthochromidis*** Bray, Cutmore & Cribb, 2018

Syn. *L. clavatum* of Bray et al. (1993), Barker et al. (1994) in part

*Type-host*: *Acanthochromis polyacanthus* (Bleeker, 1855), Spiny chromis (Pomacentridae).

*Type-locality*: Off Heron Island, southern Great Barrier Reef, Australia.

*Other locality*: Off Lizard Island, northern Great Barrier Reef, Australia.

*Records*: Bray et al. (1993); Barker et al. (1994); Bray et al. (2009); Bray et al. (2018b).

### New material

*Known host*: *A. polyacanthus.*

*Known localities*: Off Heron Island; off Lizard Island.

*Site in host*: Intestine.

*Abundance and prevalence*: Off Heron Island: six specimens from two of 15 *A. polyacanthus*. Off Lizard Island: 12 specimens from eight of 15 *A. polyacanthus*.

*Voucher material*: Five specimens (QM G240049–53), including two hologenophores.

*Representative DNA sequences*: ITS2 rDNA, two identical sequences; *cox*1 mtDNA, six sequences (four submitted to GenBank; see Table 2 for GenBank accession numbers).

### Remarks

In the present study, specimens of *L. acanthochromidis* were collected from the type-host in two known localities, off Heron and Lizard Islands. The new specimens from each locality are morphologically similar but show slight variation in some morphometrics, i.e., egg length [47–56 (51.2) *vs* 49–68 (55)] and body size [973–1,364 (1,165) *vs* 914–1,510 (1,194)]. However, comparable levels of morphological variation were also reported by Bray et al. (2018b). Newly generated *cox*1 sequence data for Heron Island specimens were identical to those published by Bray et al. (2018b). New sequences for Lizard Island specimens show that this species exhibits a strong intraspecific genetic variation that is structured geographically. Infection prevalences reported here differed from those reported by Bray et al. (2018b) in that fewer *A. polyacanthus* were infected at Heron Island (two of 15 *vs* 21 of 65) and more were infected at Lizard Island (eight of 15 *vs* 21 of 74).

***Lepotrema adlardi*** (Bray, Cribb & Barker, 1993) Bray & Cribb, 1996

Syn. *Lepocreadium adlardi* Bray, Cribb & Barker, 1993

*Type-host*: *Abudefduf bengalensis* (Bloch), Bengal sergeant (Pomacentridae).

*Type-locality*: Off Heron Island, southern Great Barrier Reef, Australia.

*Other localities*: Off Lizard Island, northern Great Barrier Reef, Australia; Ningaloo Reef, Western Australia, Australia.

*Records*: Bray et al. (1993); Barker et al. (1994); Bray et al. (2018b).

### New material

*Known host*: *A. bengalensis*.

*Known localities*: Off Heron Island; off Lizard Island.

*Site in host*: Intestine.

*Abundance and prevalence*: Off Heron Island: nine specimens from four of seven *A. bengalensis*. Off Lizard Island: six specimens from three of five *A. bengalensis*.

*Voucher material*: Four specimens (QM G240054–7), including two hologenophores.

*Representative DNA sequences*: *cox*1 mtDNA, three sequences (See Table 2 for GenBank accession numbers).

### Remarks

New specimens of *L. adlardi* were collected from *A. bengalensis* at two known localities, Heron Island and Lizard Island. New *cox*1 sequences show no geographical structuring between these sites. In the present study, prevalences were similar to those reported by Bray et al. (2018b), who reported 20 of 43 *A. bengalensis* from off Heron Island and two of five from off Lizard Island to be infected.

***Lepotrema amblyglyphidodonis*** Bray, Cutmore & Cribb, 2018

Syn. *Lepocreadium* sp. of Bray et al. (1993) and Barker et al. (1994)

*Type*-*host*: *Amblyglyphidodon curacao* (Bloch), Staghorn damselfish (Pomacentridae).

*Type*-*locality*: Off Heron Island, southern Great Barrier Reef, Australia.

*Other host*: *Amphiprion akindynos* Allen, Barrier Reef anemonefish (Pomacentridae).

*Records*: Bray et al. (1993); Barker et al. (1994); Bray et al. (2018b).

### New material

*New hosts*: *Plectroglyphidodon dickii* (Liénard), Dick’s damsel; *Stegastes apicalis* (De Vis), Australian gregory (Pomacentridae).

*Known hosts*: *A. curacao*; *Amphiprion akindynos*.

*Known locality*: Off Heron Island.

*Site in host*: Intestine.

*Abundance and prevalence*: Five specimens from three of 23 *A. curacao*; one specimen from one of seven *Amphiprion akindynos*; one specimen from one of three *P. dickii*; one specimen from one of 55 *S. apicalis*.

*Voucher material*: Two specimens (QM G240058–9), both hologenophores.

*Representative DNA sequences*: ITS2 rDNA, one sequence; *cox*1 mtDNA, two identical sequences (See Table 2 for GenBank accession numbers).

### Remarks

The specimens collected in the present study were genetically identical to those of Bray et al. (2018b). Although the single specimen collected from *S. apicalis* resembles *L. monile*, which was reported in the same host by Bray et al. (2018b), the new specimen differs slightly by having a muscular pad at the distal metraterm. The infection prevalences reported here are low and similar to those of Bray et al. (2018b), who reported low infection prevalences in *A. curacao* (five of 71) and *Amphiprion akindynos* (one of seven).

### Phylogenetic results

**Fig. 3:**
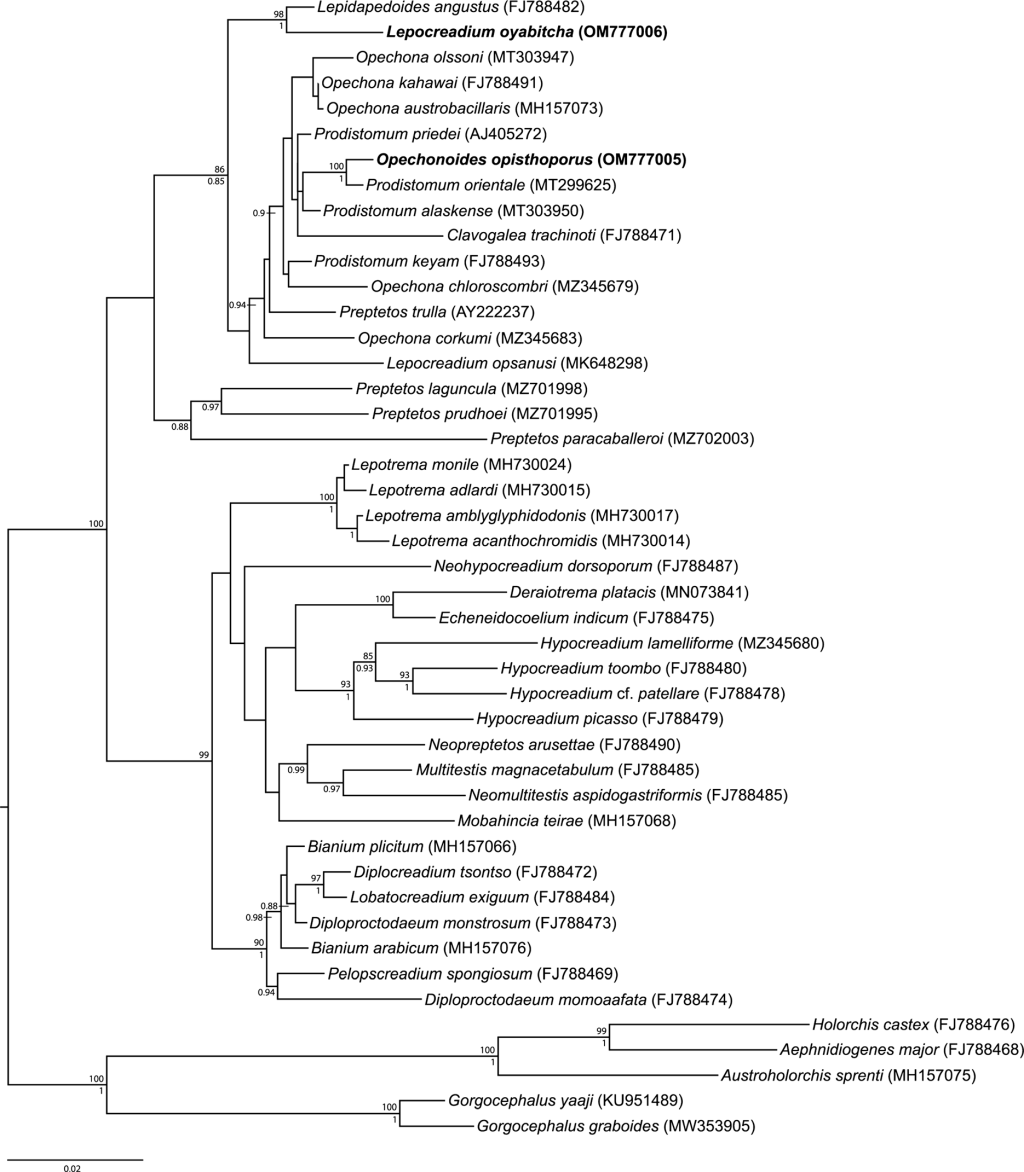
Inferred relationships of *Lepocreadium oyabitcha* and *Opechonoides opisthoporus ***n. sp.** within the Lepocreadiidae based on a maximum likelihood analysis of the 28S rDNA dataset. The newly generated sequences are in bold. Bootstrap support values are shown above the nodes, and where relationships were replicated in the Bayesian inference analysis, posterior probabilities are shown below. Support values below 0.85 or 85 are not shown. Outgroup taxa are species of the Aephnidiogenidae and Gorgocephalidae. The scale bar indicates the number of substitutions per site

Maximum likelihood and Bayesian inference analyses of the partial 28S sequences produced phylogenetic trees with almost identical topologies but varying levels of nodal support (Fig. 3). While both analyses show strong support for two major clades within the Lepocreadiidae, the key difference between the two phylogenies is the placement of *Mobahincia teirae* Bray, Cribb & Cutmore, 2018 and *Neohypocreadium dorsoporum* Machida & Uchida, 1987 in the first major clade. In the maximum likelihood analysis, *M. teirae* is sister to a clade containing *Neomultitestis aspidogastriformis* Bray & Cribb, 2003, *Multitestis magnacetabulum* Mamaev, 1970 and *Neopreptetos arusettae* Machida, 1982. *Neohypocreadium dorsoporum* is sister to the previously mentioned clade, four species of *Hypocreadium* Ozaki, 1936, *Echeneidocoelium indicum* Simha & Pershad, 1964 and *Deraiotrema platacis* Machida, 1982. In the Bayesian inference analysis, *M. teirae* and *N. dorsoporum* formed a polytomy with all the taxa in this first clade. In both analyses, *Lepocreadium oyabitcha* and *Opechonoides opisthoporus* **n. sp.** fall within the second major clade. *Opechonoides opisthoporus* **n. sp.** is sister to *Prodistomum orientale* (Layman, 1930) Bray & Gibson, 1990 (with strong support) and both formed a polytomy with *P. priedei* Bray & Merrett, 1998, *P. alaskense* (Ward & Fillingham, 1934) Bray & Merrett, 1998 and *Clavogalea trachinoti* (Fischthal & Thomas, 1968) Bray & Gibson, 1990. *Lepocreadium oyabitcha* is sister to *Lepidapedoides angustus* Bray, Cribb & Barker, 1996 (with strong support), and is separated from *Lepocreadium opsanusi* Sogandares & Hutton, 1960.

## Discussion

### Phylogeny

The phylogeny and systematics of the Lepocreadioidea have been extensively reviewed by Bray et al. (2009), Bray & Cribb (2012) and Bray et al. (2018a); these studies show that the Lepocreadiidae divides into two major clades, Clade VII and Clade VIII of Bray et al. (2009). Our analyses demonstrate that *Lepocreadium oyabitcha* and *Opechonoides opisthoporus* **n. sp.** both fit into Clade VII, which consists of multiple polyphyletic genera. The genus *Lepocreadium* appears to be polyphyletic, with *L. oyabitcha* separated from *L. opsanusi*, and sister to *Lepidapedoides angustus.* With genetic data for only two of 27 recognised *Lepocreadium* species and one of seven recognised *Lepidapedoides* species (WoRMS Editorial Board, 2021), it is difficult to interpret this relationship, especially given that *L. oyabitcha* and *Lepidapedoides angustus* are quite distinct morphologically and infect hosts from different families (*L. angustus* infects species of the Serranidae). It is noteworthy, however, that both species infect the gall bladders of their hosts (Bray et al., 1996; Justine et al., 2010). The only other species of *Opechonoides*, *O. gure*, was described from a single specimen collected from the large-scale blackfish, *Girella punctata* Gray (Kyphosidae) off Japan (Yamaguti, 1940), and, to our knowledge, it has not been reported elsewhere and no genetic data exist. Although the analyses show that *O. opisthoporus* **n. sp.** is embedded within a clade of *Prodistomum* species, it is clear that at best *Prodistomum* is paraphyletic and requires molecular study of its type-species *P. gracile* Linton, 1910.

### Mitochondrial DNA

Interestingly, the two species of *Lepotrema* collected from two localities (Heron Island and Lizard Island), showed differing patterns of genetic variation. Based on *cox*1 sequences, specimens of *L. acanthochromidis* have a high level of intraspecific variation between samples collected from the two sites, differing by a minimum of 17 base pairs. A similar level of intraspecific variation was reported for specimens of *L. hemitaurichthydis* Bray, Cutmore & Cribb, 2018, which differed by 14 base pairs between samples from French Polynesian waters and off Palau (Bray et al., 2018b). Comparable intraspecific *cox*1 variation has been reported for several trematode species in Indo-Pacific fishes: six species of the Aporocotylidae Odhner, 1912 (see Cutmore et al., 2021), three species of the Gorgocephalidae Manter, 1966 (see Huston et al., 2021), three species of the Lepocreadiidae (see Bray et al., 2018b; 2022) and seven species of the Monorchiidae Odhner, 1911 (see McNamara et al., 2014). Conversely, sequences of *L. adlardi* collected from off Heron and Lizard Islands differed by up to two base pairs, but with no geographical structuring. A similarly low level of variation was reported for *L. cirripectis* Bray, Cutmore & Cribb, 2018 (see Bray et al., 2018b). Several trematode species also show little to no differences in *cox*1 sequences from sites at least as distant as Heron and Lizard Islands: one species of the Aporocotylidae (see Cutmore et al., 2021), two species of the Fellodistomidae Nicoll, 1909 (see Cribb et al., 2021), five species of the Lepocreadiidae (see Bray et al., 2018b; 2022) and three species of the Monorchiidae (see McNamara et al., 2014). These varying patterns of intraspecific genetic variation suggest that trematodes are likely differentially affected by geographical or host-related factors which either limit or benefit their dispersal; however, no synthesis of explanation has emerged so far.

In the case of *L. acanthochromidis*, it is possible that host-related factors play a role in the high level of intraspecific genetic variation that we see in the *cox*1 region [and the 28S region (Bray et al., 2018b)]. Populations of the host, *Acanthochromis polyacanthus*, exhibit strong levels of phenotypic and genetic variation across the GBR (Doherty et al., 1994; Planes et al., 2001; van Herwerden & Doherty, 2006). This species is unique among GBR pomacentrids as it lacks a dispersive pelagic larval stage (Robertson, 1973) and adults exhibit low rates of migration between reefs (Miller-Sims et al., 2008). Populations separated by large areas of deep water (e.g., the Swain Reefs and the Capricorn Bunker Group reefs, which are approximately 150 km apart) show strong levels of genetic structuring, whereas populations on patch reefs separated by shallow water (e.g., Heron Island reefs and Sykes reefs which are less than 2 km apart) show no population differentiation (Miller-Sims et al., 2008). Furthermore, groups of juvenile *A. polyacanthus* only travel between 50–100 metres between patch reefs (Kavanagh, 2000). These low rates of movement may be effective in limiting the gene flow of *L. acanthochromidis,* as parasite gene flow is expected to reflect or be determined by hosts with the highest dispersal ability (Prugnolle et al., 2005). Indeed, it is possible that *L. acanthochromidis* relies on intermediate hosts for dispersal, as there is evidence that gelatinous organisms (with high dispersal capabilities) are used by another species of *Lepotrema* (Kondo et al., 2016). However, given the distinct geographical genetic structuring, it is likely that *L. acanthochromidis* uses intermediate host taxa that also have limited dispersal capabilities. It is plausible that historic patterns of sea level resulting in the connectivity (via shallow water ‘crossings’) of distant present-day reefs allowed for the dispersal of *A. polyacanthus* (see Miller-Sims et al., 2008), *L. acanthochromidis* and the latter’s intermediate hosts. With changes in sea level, host and trematode populations have become isolated and likely show variation within the *cox*1 region due to the non-recombinant nature of mitochondrial genes (Morgan-Richards et al., 2017).

### Host-specificity

The new collections show that *Lepotrema amblyglyphidodonis*, *Lepocreadium oyabitcha* and *Opechonoides opisthoporus* **n. sp.** all exhibit stenoxenic host-specificity (Miller et al., 2011); however, each pattern is quite distinct. *Lepocreadium oyabitcha* has been reported from species of a single genus (*Abudefduf*), *Lepotrema amblyglyphidodonis* has been reported from four species belonging to four genera (*Amblyglyphidodonis*, *Amphiprion*, *Plectroglyphidodon* and *Stegastes*), and *O. opisthoporus* **n. sp.** has been reported from 12 species belonging to seven genera (*Abudefduf*, *Amphiprion*, *Neoglyphidodon*, *Neopomacentrus*, *Plectroglyphidodon*, *Pomacentrus* and *Stegastes*). Such stenoxenicity is also exhibited by *L. monile*, which has been reported from four species belonging to two genera (*Pomacentrus* and *Stegastes*). Of the remaining pomacentrid-infecting lepocreadiids, three are oioxenic (*L. acanthochromidis*, *L. adlardi* and *Lepocreadium sogandaresi*) and one is euryxenic (*Lepotrema clavatum*). However, as Bray et al. (2018b) noted, it is unlikely that the reported euryxenicity of *L. clavatum* is valid, given the strict oioxenic-stenoxenic patterns of host-specificity seen for species of *Lepotrema* specifically, and other lepocreadiids generally.

Infections of *O. opisthoporus* **n. sp.** were never common in any pomacentrid species. They were most frequent in species of *Abudefduf* and *Pomacentrus*; only single species of the other five genera were infected. However, this could be an artefact of our sampling efforts; species of *Abudefduf* and *Pomacentrus* make up over 50% of the pomacentrids that we have examined. Given that we have collected *O. opisthoporus* **n. sp.** in other pomacentrid genera, albeit at lower prevalences and abundances, it is likely that with further sampling, more pomacentrid hosts will be discovered. In contrast, the host range of *Lepocreadium oyabitcha* is unlikely to increase to include other pomacentrid genera, as our current collection records indicate that this species is restricted to species of *Abudefduf* only. Although the second intermediate host of *L. oyabitcha* remains unknown, we have examined multiple pomacentrid species that share a similar habitat and diet (planktonic organisms and algae) without finding infections of *L. oyabitcha*. It is possible that the restriction of *L. oyabitcha* to species of *Abudefduf* is related to the size of these fishes and the trematode itself. *Lepocreadium oyabitcha* is substantially larger than other pomacentrid-infecting lepocreadiids and its size may restrict it from infecting the gall bladder of small pomacentrid species.

### Geographical distribution

In the present study, specimens of *Lepotrema acanthochromidis*, *L. adlardi* and *O. opisthoporus* **n. sp.** were collected from off both Heron and Lizard Islands, which are almost 1,200 km apart. Bray et al. (2018b) also reported the same distribution for *L. acanthochromidis*, *L. adlardi* (with an additional locality: Ningaloo Reef in Western Australia) and *L. monile*. *Lepotrema amblyglyphidodonis* has only been found off Heron Island; however, the southern restriction of this species in the GBR may be an artefact of our sampling. To date, we have examined over 160 individuals of the host species, *A. curacao*, *Amphiprion akindynos*, *P. dickii* and *S. apicalis* at Heron Island whereas in comparison, fewer than 70 individuals have been examined at Lizard Island. Given the relatively broad geographical distribution of the other *Lepotrema* species, it is plausible that more sampling off Lizard Island and elsewhere will increase the geographic range of *L. amblyglyphidodonis*. In contrast, it is unlikely that the distribution of *Lepocreadium oyabitcha* includes Heron Island or Moreton Bay, as we have sampled extensively for species of *Abudefduf* in those regions without detecting it.

### Second intermediate hosts

The finding of metacercariae of *O. opisthoporus* **n. sp.** in ctenophores represents the first report of intermediate hosts for a species of *Opechonoides* and indeed for pomacentrid-infecting lepocreadiids. Several studies have reported gelatinous organisms as second intermediate hosts of other lepocreadiids, including species of *Lepocreadium*, *Lepotrema* and *Opechona* Looss, 1907 (see Stunkard, 1972, 1980; Martorelli, 2001; Ohtsuka et al., 2010; Browne et al., 2020). Interestingly, adult *O. opisthoporus* **n. sp.** have been found only in GBR pomacentrid fishes, whereas the infections of metacercariae were found in ctenophores collected as far south as off the coast of Tasmania, more than 2,000 km south of the closest known locality for the adults. We can speculate that infected ctenophores are dispersed great distances by the southward flowing East Australian Current or that infections in southern fishes have not yet been detected due to limited sampling. The dispersal capabilities of ctenophores likely influences the distribution of *O. opisthoporus* **n. sp.** and explains the lack of genetic variation seen for specimens collected from off Heron and Lizard Islands. Given that *Pukia falcata*, and species of *Bolinopsis* L. Agassiz and *Ocyropsis* Mayer have been reported at various locations along the eastern coast of Australia (Gershwin et al., 2010), it is plausible that they act as intermediate hosts on the GBR. Whilst pomacentrids are not generally known to feed on ctenophores, most are at least partly planktivorous (Pratchett et al., 2016; Cowan et al., 2019; Emslie et al., 2019) and so may well consume ctenophores; the low prevalence and intensity of infection of *O. opisthoporus* **n. sp.** in pomacentrids suggests that the consumption may be opportunistic. As other lepocreadiid metacercariae collected from gelatinous host organisms have been linked to definitive hosts that would be expected to consume gelatinous organisms (Ohtsuka et al., 2010), this study has identified a potentially novel trophic link between pomacentrid fishes and ctenophores, highlighting the potential of parasites as novel trophic tracers.

## Data Availability

The data that support the findings of this study are available from the corresponding author upon reasonable request.
